# Dose of early intervention treatment during children’s first 36 months of life is associated with developmental outcomes: an observational cohort study in three low/low-middle income countries

**DOI:** 10.1186/1471-2431-14-281

**Published:** 2014-10-25

**Authors:** Jan L Wallander, Fred J Biasini, Vanessa Thorsten, Sangappa M Dhaded, Desiree M de Jong, Elwyn Chomba, Omrana Pasha, Shivaprasad Goudar, Dennis Wallace, Hrishikesh Chakraborty, Linda L Wright, Elizabeth McClure, Waldemar A Carlo

**Affiliations:** Psychological Sciences and Health Sciences Research Institute, University of California, Merced, CA USA; Sparks Clinics and Department of Psychology, University of Alabama at Birmingham, Birmingham, AL USA; Department of Statistics and Epidemiology, RTI International, Durham, NC USA; KLE Jawaharlal Nehru Medical College, Belgaum, India; University of Massachusetts Amherst, Amherst, MA USA; University of Zambia, Lusaka, Zambia; Aga Kahn University Medical College, Karachi, Pakistan; University of South Carolina, Columbia, SC USA; the Eunice Kennedy Shriver National Institute of Child Health and Human Development (NICHD), Bethesda, MD USA; Department of Pediatrics, University of Alabama at Birmingham, Birmingham, AL USA

**Keywords:** Treatment dose, Early developmental intervention, Neurodevelopmental disability, Birth asphyxia, Developing countries

## Abstract

**Background:**

The positive effects of early developmental intervention (EDI) on early child development have been reported in numerous controlled trials in a variety of countries. An important aspect to determining the efficacy of EDI is the degree to which dosage is linked to outcomes. However, few studies of EDI have conducted such analyses. This observational cohort study examined the association between treatment dose and children’s development when EDI was implemented in three low and low-middle income countries as well as demographic and child health factors associated with treatment dose.

**Methods:**

Infants (78 males, 67 females) born in rural communities in India, Pakistan, and Zambia received a parent-implemented EDI delivered through biweekly home visits by trainers during the first 36 months of life. Outcome was measured at age 36 months with the Mental (MDI) and Psychomotor (PDI) Development Indices of the Bayley Scales of Infant Development-II. Treatment dose was measured by number of home visits completed and parent-reported implementation of assigned developmental stimulation activities between visits. Sociodemographic, prenatal, perinatal, and child health variables were measures as correlates.

**Results:**

Average home visits dose exceeded 91% and mothers engaged the children in activities on average 62.5% of days. Higher home visits dose was significantly associated with higher MDI (mean for dose quintiles 1–2 combined = 97.8, quintiles 3–5 combined = 103.4, *p* = 0.0017). Higher treatment dose was also generally associated with greater mean PDI, but the relationships were non-linear. Location, sociodemographic, and child health variables were associated with treatment dose.

**Conclusions:**

Receiving a higher dose of EDI during the first 36 months of life is generally associated with better developmental outcomes. The higher benefit appears when receiving ≥91% of biweekly home visits and program activities on ≥67% of days over 3 years. It is important to ensure that EDI is implemented with a sufficiently high dose to achieve desired effect. To this end groups at risk for receiving lower dose can be identified and may require special attention to ensure adequate effect.

## Background

Programs of early developmental intervention (EDI) implemented in the first years of life in children born with, or at risk for, neurodevelopmental disability have been shown to improve cognitive developmental outcomes and consequently, their quality of life. EDI includes various activities designed to enhance a young child’s development, directly via structured experiences and/or indirectly through influencing the care giving environment [1]. The positive effects of EDI on early child development have been reported in numerous controlled trials in high-income countries [2, 3], which have been confirmed through meta-analyses [4, 5] and expert reviews [6–8]. Several trials of EDI with risk groups of infants and young children have also been conducted in low or low-middle income countries (L/LMIC), which have also documented positive effects on child development, by itself or in combination with nutritional supplementation [9–16].

The involvement of parents in EDI is critical for achieving positive outcomes [1, 17–19], which can be optimized by implementing EDI through home visits by a parent trainer. This modality also matches well the circumstances of many L/LMIC where families often live far away from or have other barriers to reach providers that could implement EDI [20]. An important aspect to determining the efficacy of EDI is the degree to which dosage impacts outcomes, and what constitutes “sufficient dosage” [21]. Sufficient dosage with regard to EDI refers to a participant receiving adequate exposure to the intervention for it to be efficacious. Program intensity, or dosage, typically is measured by the quantity and quality the intervention actually achieved when implemented [21, 22], although it ideally should be determined based on the needs of the population at hand [23]. Common indicators of dosage for EDI include amount of time spent in a child development center, number of home visits completed by a specialist training a parent and/or engaging the child, or some indication of parent engagement in the EDI.

Whereas there is more information linking outcomes with treatment dose for pre-school programs [21, 22], despite its importance few studies of EDI implemented in the first three years of life have conducted such analyses. A few previous studies generally indicate that children who receive more exposure to EDI display greater improvements in their cognitive development compared to those who receive less, even when differences in exposure were modest. Specifically, children who received EDI (home and center based) for more than 400 days, through age 3, exhibited significant improvements in cognitive development, while smaller but similar effects were evident among children who received treatment between 350 and 400 days [24]. Another study reported that optimal cognitive development of children in EDI was not associated with their background characteristics, such as birth weight or maternal education, but with three aspects related to treatment dosage: number of home visits received, days attending child care, and number of parent meetings attended [18].

However these studies as well as the broader discussions of implementation quality have focused on programs conducted in the United States [21, 22]. The applicability of this information to L/LMIC contexts is unclear at present. The only EDI treatment dose study conducted in a L/LMIC that we are aware of showed that, as the frequency of home visits increased from none, through monthly, biweekly, and weekly, developmental gains at 30 months of age increased as well [25]. Given the potential for EDI to significantly impact the development of children, and therefore the economic development of nations in the long-term [26], it will be important more broadly to examine treatment dose in L/LMIC to inform the implementation of such efforts on a larger scale.

Parents may vary in their level of participation in home visit EDI programs due to a variety of factors. Previous research has indicated higher treatment dose among families participating in EDI who have better financial and social resources [20, 27–30]. Perinatal, neonatal, and other child health characteristics might also predict treatment dose for an intervention intending to promote the child’s development. Yet, studies that have examined both social and health predictors of EDI treatment dose are rare and have not considered a broad range of possible predictors [15]. It is important to examine various such factors in L/LMIC because they can identify processes that may influence parents’ adherence with EDI and those who may need additional support.

In light of these gaps in our understanding, the aim of the current study was to determine (1) whether there is a dose effect in a home visiting EDI implemented in three L/LMIC and (2) what sociodemographic and health factors are associated with variation in treatment dose. We examined two indicators of dose of EDI. As in previous studies, the number of home visits completed over the course of the EDI was measured. Another important treatment element is the extent to which parents implement the assigned developmental activities with the child during the time between home visits, which we refer to as the program implementation dose. Despite its logical importance to the success of home visiting EDI, we are not aware that parent program implementation dose has been examined in EDI. We hypothesize that increased dose as measured by either indicator will be associated with better developmental outcomes from EDI when implemented in three L/LMIC.

## Methods

Data used to examine the association between treatment adherence and developmental outcomes are from one of the conditions of the Brain Research to Ameliorate Impaired Neurodevelopment - Home-based Intervention Trial (BRAIN-HIT), a randomized controlled trial (RCT) detailed elsewhere (clinicaltrials.gov ID# NCT00639184) [31, 32]. Implemented in rural communities of India, Pakistan, and Zambia, the overall aim of BRAIN-HIT was to evaluate the efficacy of an EDI program on the development of children in L/LMIC who are at-risk for neurodevelopmental disability due to birth asphyxia that required resuscitation. A group of children who did not require resuscitation at birth was evaluated using the same protocol to compare the efficacy of the EDI in those with and without birth asphyxia.

As detailed elsewhere [32, 33], mental development at 36 months of age was better in children with birth asphyxia who had received the EDI compared with those in the control condition (effect size = 4.6 points on the standardized scale from the Bayley Scales of Infant Development, see below), but there was no difference between trial conditions in the children without birth asphyxia. Psychomotor development was likewise higher in the EDI group, in this case for both the children with (effect size = 5.4) and without (effect size = 6.1) birth asphyxia, compared to those in the control condition. The issue of the effect of treatment dose on development is only relevant for the active EDI condition, and not the comparison condition, which intended to control for placebo, observation, and time effects and lacked a theoretically based developmental intervention. Therefore, only data from those randomized to receive EDI were analyzed in the present research, making this an observational study of that cohort. BRAIN-HIT was approved by the Institutional Review Board at each site and was conducted in accord with prevailing ethical principles.

### Study population

Infants with birth asphyxia (resuscitated) and infants without birth asphyxia or other perinatal complications (non-resuscitated), born from January 2007 through June 2008 in rural communities in three sites in India, Pakistan and Zambia, were matched for country and chronological time and randomly selected from those enrolled in the First Breath Trial [34]. Infants were screened for enrollment into the BRAIN-HIT during the 7-day follow-up visit after birth [31], and were ineligible if: (1) birth weight was less than 1500 grams, (2) neurological examination at seven days of age (grade III by Ellis classification) [35]**,** was severely abnormal (because they were not expected to benefit from EDI), (3) mother was less than 15 years old or unable/unwilling to participate, or (4) mother was not planning to stay in the study area for the next three years. Birth asphyxia was defined as the inability to initiate or sustain spontaneous breathing at birth using WHO definition (biochemical evidence of birth asphyxia could not be obtained in these settings) [36]. A list of potential enrollees was distributed to the investigators in each country to obtain written consent for the study, which was obtained during the second week after birth and before randomization to intervention conditions of the BRAIN-HIT.

### Intervention procedures

Investigators at each research site selected EDI parent trainers who were trained in an initial 5-day workshop, which was led by the same experts at each research site. A second workshop was conducted before participating children began to reach 18 months of age to adapt the approach to children up to 36 months, again conducted by the same experts at each site. To maintain quality of implementation, the trainers were supervised with observations during actual home visits and constructive feedback was provided on a regular basis.

Each parent–child pair was assigned to the same trainer throughout the trial whenever possible, who was scheduled to make a home visit every two weeks over the 36-month trial period. As elaborated elsewhere [31, 32], the trainer presented one or two playful learning activities during each visit targeting developmentally appropriate milestones. These activities cover a spectrum of abilities across the cognitive, social and self-help, gross and fine motor, and language domains. The parent practiced the activity in the presence of the trainer who provided feedback. Cards depicting the activities were then left with the parent, who was encouraged to apply the activities in daily life with the child until the next home visit. The trainer introduced new activities in subsequent visits to enhance the child’s developmental competencies.

### Treatment dose indicators

Two indicators of treatment dose were calculated. *Home visit dose* was measured based on each parent trainer keeping a record of visit dates. Following the first visit, visits were scheduled to occur every two weeks until the completion of the trial. A home visit was completed on schedule if it occurred within its assigned two week window following the preceding visit. We calculated the percentage of scheduled home visits completed for each participant for the full 36-month trial. The reason for each missed visit was coded as due to illness, weather, death in family, refusal, child or mother unavailable for another reason, parent trainer schedule conflict, and other reasons.

*Program implementation dose* was measured based on maternal report obtained by the trainer at each home visit of the proportion of days the assigned activities had been implemented since the previous visit. First, the number of days between subsequent completed visits was calculated (Y_n_). If the time between two home visits extended beyond 30 days, a maximum of 30 days was used. Program implementation credits were assigned for the time period between visits based on the mother’s report of implementation of activities, as follows: “not at all” (credit_n_ = 1), “about one-quarter of days or less” (credit_n_ = Y_n_*.25), “about one-half of days” (credit_n_ = Y_n_*.50), “about three-quarters of days” (credit_n_ = Y_n_*.75), and “almost every day or more” (credit_n_ = Y_n_). The credits were then added together over the trial period, divided by the number of possible credits, and multiplied by 100. Thus, this score estimates the percent of days between each home visit that the mother reported implementing child stimulation activities. As an additional descriptive measure of treatment dose**,** the parent trainer was surveyed at the conclusion of the study to estimate how often the activities had been implemented between the home visits, using a five-point scale (from “never” to “always”).

### Developmental outcome measures

The Bayley Scales of Infant Development – II (BSID) [37] was selected as the main outcome measure for this trial because it has been used extensively in various L/LMIC. The BSID underwent pilot-testing at each site to verify validity in the local context and a few items were slightly modified to make it more culturally appropriate (e.g., image of a sandal instead of a shoe). Evaluators across the sites were trained to standards in joint 4-day workshops conducted by experts before each yearly evaluation. The BSID was administered directly to each child by certified study evaluators, who were masked to the children’s birth history and randomization, in the appropriate language with standard material. Both the Mental Developmental Index (MDI) and Psychomotor Developmental Index (PDI) were used to measure developmental outcomes. Scores from the 36-month assessment, obtained just after the completion of the EDI, were used in this analysis as an indicator of treatment outcome.

### Health and sociodemographic measures

Perinatal and neonatal health variables were obtained from records kept by the FIRST BREATH Trial [34]: child gender, birth weight (1500 g-2499 g, 2500 g-2999 g, 3000 + g), gestational age (28–36 weeks, 37+ weeks), number of prenatal visits (0, 1–3, 4+), and parity. Additional child health variables obtained as part of this trial at 12 months of age included weight for age/sex (<5th, 5th-14th, 15th + percentile) and complete immunization status.

Family demographic variables were obtained at enrollment in BRAIN-HIT using a structured parent interview: maternal age, education (none and illiterate, none but literate or primary, literate with some secondary), family assets and home living standard. The presence of 11 family assets (e.g., radio, refrigerator, bicycle) were tallied as a Family Resources Index and classified into three levels (0–1, 2–4, 5+). A Home Living Standard Index was calculated based on seven indicators (e.g., home building material, water source, type of toilet) and classified into three levels (0–4, 5–7, 8+). A socio-economic status (SES) measure was used to classify participants into three groups (quintile 1–3, 4, 5) [38].

### Statistical analysis

Descriptive statistics were computed for child health and family demographic characteristics, treatment dose indicators (home visits dose and protocol implementation dose), and developmental outcomes (MDI and PDI at 36-months) for all individuals randomized to receive EDI. Child health and demographic characteristics were summarized separately for those randomized to receive EDI and included in the treatment dose analysis and those who were excluded from this analysis, and differences in mean values for continuous variables were tested using t-tests and categorical measures were tested using chi-square and Fisher exact tests. A Pearson correlation statistic was computed between the treatment dose characteristics.

#### Aim 1

In the absence of established criteria for adequate treatment dose for EDI and to determine where the effectiveness of the intervention may plateau, both treatment dose indicators were divided into quintiles. Those in quintile 1 had lowest dose and those in quintile 5 had the highest dose of the indicator in question. Descriptive statistics for the 36-month MDI and PDI were calculated for each quintile. General linear models were used to evaluate the associations of treatment dose quintile with 36-month MDI and PDI. In addition to the treatment dose indicator in question, covariates of interest included resuscitation status at birth, 12-month MDI or PDI, and site. If the omnibus 4-degree of freedom test for either MDI or PDI provided evidence of significant differences across quintiles of treatment dose, step-down tests were used to evaluate where those differences occurred.

#### Aim 2

To evaluate associations with treatment dose, initially all sociodemographic and child health variables and trial location were entered into linear regression models separately to predict both treatment dose variables. Selected for entry in multivariable models were variables that demonstrated *P* ≤ 0.20 in univariate association with the adherence variable in question when either adjusted by location alone or location and the variable by location interaction. We employed backward elimination with an alpha of 0.20 to choose the final models.

## Results

### Study sample composition

**Figure 1:**
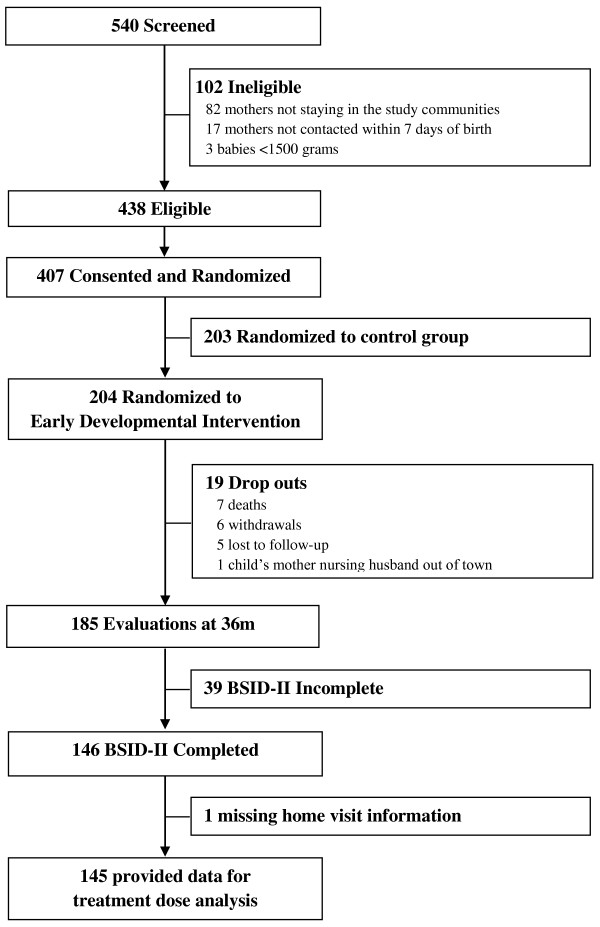
**Study flow chart.**

The sample size was determined to provide adequate power to test EDI treatment efficacy, the primary aim of BRAIN-HIT. As outlined in Figure 1, of 540 births screened from January 2007 through June 2008, 438 (81% of screened) were eligible. Only 3 infants were ineligible due to low birth weight or neurological exam, with the remaining 99 being due to mothers not being able to commit to staying in the study communities or could not be reached for screening within 7 days of birth. Informed consent was obtained for 407 (93% of eligible; 165 resuscitated, 242 not resuscitated) who were randomized into either EDI or a control intervention [20]. The 204 assigned to receive EDI (50.1% of those randomized) are relevant for this study, of whom 145 (71.1% of those assigned to EDI) were included in this analysis (Table 1). These participants had mean = 36.8 (range = 35-41) months of age at the time of the developmental assessment.

**Table 1:** **Child health and family demographic characteristics of study sample**

Characteristic ^a^	Inclusion in analysis - n (%)		Total	p-value ^b^
	Yes	No		
Enrolled - N	145	59	204	--
Male gender- n/N (%)	78/141 (55.3)	34/59 (57.6)	112/200 (56.0)	0.7643
Birth weight (grams) - N	139	59	198	0.1109
1500 - 2499	30 (21.6)	13 (22.0)	43 (21.7)	
2500 - 2999	51 (36.7)	30 (50.8)	81 (40.9)	
≥ 3000	58 (41.7)	16 (27.1)	74 (37.4)	
Gestational age-mean (std)	37.9 (2.0)	37.8 (1.9)	37.9 (2.0)	0.7804
Preterm (<37 mos.) - n/N (%)	40/142 (28.2)	16/59 (27.1)	56/201 (27.9)	0.8798
Resuscitated - n/N (%)	59/145 (40.7)	19/59 (32.2)	78/204 (38.2)	0.2581
Prenatal care visits-N	145	59	204	**0.0151**
0	35 (24.1)	4 (6.8)	39 (19.1)	
1-3	48 (33.1)	22 (37.3)	70 (34.3)	
4 or more	62 (42.8)	33 (55.9)	95 (46.6)	
Weight for age and sex at 12 mos-N	137	43	180	**0.0407**
<5th % tile for age in months	40 (29.2)	20 (46.5)	60 (33.3)	
5th-14th % tile for age in months	16 (11.7)	7 (16.3)	23 (12.8)	
> = 15th % tile for age in months	81 (59.1)	16 (37.2)	97 (53.9)	
Immunization complete 12 mos-n/N (%)	106/142 (74.6)	41/43 (95.3)	147/185 (79.5)	**0.0032**
Maternal Age (Years)- Mean (Std)	25.5 (5.7)	24.3 (4.0)	25.1 (5.3)	0.0844
Maternal schooling completed-N	136	58	194	0.5144
None and illiterate	68 (50.0)	25 (43.1)	93 (47.9)	
Literate or primary schooling	36 (26.5)	20 (34.5)	56 (28.9)	
Literate and some secondary schooling	32 (23.5)	13 (22.4)	45 (23.2)	
Parity (including child enrolled in study) - Mean (Sd)	3.1 (2.2)	2.4 (1.3)	2.9 (2.0)	**0.0110**
Family Resources Index (# items present in home) - N	145	59	204	**<.0001**
0-1	55 (37.9)	11 (18.6)	66 (32.4)	
2-4	49 (33.8)	40 (67.8)	89 (43.6)	
5+	41 (28.3)	8 (13.6)	49 (24.0)	

^a^Measured at enrollment unless otherwise indicated.

^b^Differences in mean values for continuous variables were tested using t-tests and categorical measures were tested using chi-square and Fisher exact tests; bold indicates significant p < .05.

Exclusions from this analysis were due to death (n = 7), withdrawal (n = 6), loss to follow up (n = 5), incomplete 36-month BSID-II (n = 39) due to administration errors, home-visit data unavailable (n = 1), or another reason (n = 1). Three children were included in the analysis who completed the 36-month evaluation but discontinued the EDI prior to the end of the study (two because the family had insufficient time to fulfill study requirements and one because the family moved). When compared to those who were included in the analysis (Table 1), children excluded (n = 59) were significantly (*p* < .05) more likely to have been less than the 5th percentile in weight and completed all immunizations at 12-months of age, and their mothers to have had prenatal care, lower parity, and more family resources.

### Description of developmental outcomes and treatment dose

The sample had an unadjusted mean (SD) MDI = 101.2 (10.4) and PDI = 106.8 (14.1) at 36-months. Average home visits dose was 91.4% over 36 months, when 8,990 visits out of 9,841 were completed on schedule every two weeks, and 95% of the participants achieved 80% or greater home visits dose. The most common reason for a missed visit was the inability to locate the mother and child at home at the scheduled time (40.3%), for example because the family was travelling away from the home or had moved temporarily. However, the second most common reason was those related to the parent trainer, such as being ill or having a conflict with another meeting (23.9%). Child or mother unavailable for other reasons (15.3%), for example because the mother was working or baby was sleeping, and weather (10.0%) were the only other reasons accounting for at least 10% of the missed visits. Mother or family directly refusing the home visit at the scheduled time was rare (2.5%).

Mothers reported engaging the child in the assigned activities on an average of 62.5% of days throughout the 36 month period. This protocol implementation dose equates to practicing the intervention activities 4.4 days per week or 674 days over the 36 month trial period. Home visits dose was modestly correlated with protocol implementation dose (*r* = 0.35). Parent trainers estimated at the end of the trial that 66.2% of families practiced the intervention “always” or “almost always” throughout the 36 months.

### Associations between treatment dose and developmental outcomes

**Figure 2:**
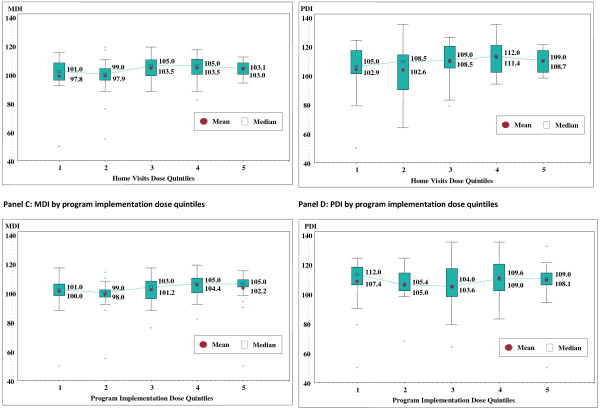
**Mental (MDI) and Psychomotor (PDI) Development Index by treatment dose quintiles.**

Higher *home visits dose* was associated with higher MDI at 36-months (Figure 2). Specifically, quintiles 1–2 mean MDI = 98, while quintiles 3–5 mean MDI = 103 (Table 2). General linear models of MDI supported this relationship when home visits dose was entered as a primary predictor and site, resuscitation status at birth, and 12-month MDI were entered as covariates (Table 2). Most notably, in the model with only home visits dose (Model 1) and the model which included site (Model 2), mean MDI for quintiles 1 and 2 was significantly lower than quintiles 3–5. A step-down test comparing mean MDI for those with home visit dose below the 40th percentile (quintiles 1 and 2) to those with home visit dose above the 40th percentile (quintiles 3–5), provided estimates of 97.8 and 103.4 (*p* = 0.0017), respectively . Adjusting by site increased the magnitude of the difference by at least 25% (96.8 vs. 103.9, *p* = 0.0005). When adjusting for 12-month MDI and the interaction between dose and 12-month MDI (Model 5), the adjusted mean scores for the dose quintiles mirrored unadjusted scores, with quintiles 1–2 consistently lower than quintiles 3–5 (p <0.0001). The lower limit for quintile 3 includes those receiving a minimum of 91% of all the planned home visits.

**Table 2:** **Treatment dose modeling results and mean mental (MDI) and psychomotor (PDI) developmental index by quintiles**

Outcome	Dose indicator	Model number	Covariates	p-values		Least squares means for quintiles				
				Dose ^a^	Covariate	Q1	Q2	Q3	Q4	Q5
MDI	Home Visits Dose	1	--	**0.0443**		97.8	97.9	103.5	103.5	103.1
		2	Site	**0.0150**	0.2159	96.3	97.0	103.3	104.4	104.2
		3	Resuscitation	0.0802	0.5155	98.1	98.1	103.5	103.4	103.2
		4	12 Mo MDI	**0.0296**	<0.0001	98.3	97.6	102.8	103.8	103.4
		5	12 Mo MDI	**<0.0001**	<0.0001	98.6	97.4	103.4	103.6	103.1
			Interaction		<0.0001					
PDI	Home Visits Dose	1	--	0.0669		102.9	102.6	108.5	111.4	108.7
		2	Site	0.0823	0.1692	102.9	102.2	109.0	112.2	108.7
		3	Resuscitation	0.1160	0.5346	103.3	102.9	108.5	111.3	108.7
		4	12 Mo PDI	0.2588	0.0024	104.2	103.0	108.4	110.2	108.1
		5	12 Mo PDI	**0.0030**	0.0421	106.5	103.3	108.5	111.0	109.4
			Interaction		0.0135					
MDI	Program Implemen-tation Dose	1	--	0.1901		100.0	98.0	101.2	104.4	102.2
		2	Site	0.2016	0.8523	100.0	98.0	101.8	104.5	102.0
		3	Resuscitation	0.2225	0.2338	100.1	98.4	101.5	104.6	102.4
		4	12 Mo MDI	0.2661	<0.0001	100.3	98.4	100.9	103.5	103.0
		5	12 Mo MDI	**0.0434**	0.0005	100.1	98.3	100.9	105.0	103.1
			Interaction		0.0641					
PDI	Program Implemen-tation Dose	1	--	0.5182		107.4	105.4	103.6	109.6	108.1
		2	Site	0.8002	0.3009	107.1	105.8	105.1	109.5	108.0
		3	Resuscitation	0.5907	0.2590	107.5	105.9	104.0	109.7	108.2
		4	12 Mo PDI	0.7654	0.0007	108.2	105.3	104.7	108.7	107.3
		5	12 Mo PDI	0.3491	0.0011	108.6	105.3	105.5	109.6	107.2
			Interaction		0.4095					

^a^Bold indicate significant *p* < .05 for the relationship between the treatment dose indicator and the developmental outcome.

Based on the same general linear model analysis (Table 2), home visit dose was not significantly associated with PDI at 36 months when considered by itself (Model 1) or when adjusted by site, resuscitation status, and 12-month PDI (Models 2–4). However, there was a positive association between home visits dose and 36-month PDI when adjusting for the 12-month PDI and its interaction with dose (Model 5). Here again, a home visit dose above the 40th percentile (quintiles 3–5) resulted in higher estimated PDI (108.5 – 111.0) compared with below this percentile (103.3 – 106.5)

Higher *program implementation dose* was associated with slightly higher MDI at 36-months compared to those with a lesser dose. Quintiles 1–2 had a mean MDI of 100 or lower, while quintiles 4–5 has a mean MDI of 102 or higher (Table 2), and the difference appears larger when considering the medians of these quintiles. In a general linear model of 36-month MDI (Table 2), program implementation dose was not a significant predictor by itself (Model 1). However, prediction of program implementation dose when adjusting for 12-month MDI and its interaction with dose (Model 5) indicated that greater dose was associated with higher MDI (adjusted mean Q1 = 100.1 vs. Q5 = 103.1, *p* = 0.0434). PDI at 36 months was not linearly associated with program implementation dose (Table 2). Rather, mean PDI across quintiles followed a U-shape with the highest mean scores for quintiles 1, 4 and 5. The lower limit for quintile 4 includes those implementing activities on 67% of days on average over the trial period.

### Factors associated with treatment dose

The following variables were associated with *home visits dose* at *P* ≤ 0.20 when either adjusted by location or by the location by variable interaction: maternal education, parity, family resources, prenatal visits, birth attendant, 1 minute Apgar, preterm birth, and child’s weight at 36-months. These variables were entered into a generalized linear model along with those interaction terms with location that were significant. After backward elimination, the final model (R^2^ = .19) included parity (82.9 ± 3.0 [adjusted mean ± standard error] with 1 child, 79.7 ± 2.8 with 2–3 children, and 90.8 ± 3.5 with 4+ children [p = 0.0382]), 1 minute Apgar (86.9 ± 2.6 for <9 and 82.0 ± 2.6 for 9+ [p = 0.1754]), location (adjusted mean ranged from 75.6 - 94.1, [p = 0.0019]), preterm [(p = 0.4571) and preterm by location interaction(p = 0.0020). There was a substantial difference in relationship to home visits dose by prematurity across location. Location A had higher dose for term children (65.8 ± 6.3 for preterm and 85.3 ± 4.0 for term). Location B had essentially the same dose between groups (92.8 ± 5.9 for preterm and 95.4 ± 2.9 for term). Location C had considerably higher dose in preterm children (90.5 ± 4.6 for preterm and 76.9 ± 3.5 for term).

The following variables were associated with *program implementation dose* at *P* ≤ 0.20 when either adjusted by location or by the location by variable interaction: home visit adherence rate, maternal education, parity, family resources, living standard index, prenatal care, 1 minute Apgar, preterm birth, and weight at birth, 12, 24, and 36 months. These variables were entered into a model along with those interaction terms with location that were significant. After backward elimination and adjusting for location, the final model (R^2^ = .25) included home visit adherence rate (a one percent increase in home visit adherence resulted in a 0.64 ± 0.18 percent increase in program implementation adherence, p = 0.0004), maternal education (70.0 ± 2.8 for secondary/university and 60.9 ± 2.4 for none/illiterate [p = 0.0400]), prenatal care (71.0 ± 2.9 for 5+ visits and 65.3 ± 3.5 for no care [p = 0.0170]), weight at 12 months (66.7 ± 1.7 for >85th percentile and 61.1 ± 2.2 for <5th percentile [p = 0.0917]), and location (adjusted mean ranged from 59.5 - 69.1, [p = 0.0019]). None of the interaction terms were retained in the final model.

## Discussion

Consistent with our hypothesis, receiving a higher dose of EDI during the first 36 months of life, as indicated by number of home visits by a parent trainer and reported implementation of program activities between these home visits, is generally associated with better developmental outcomes at 36 months of age. This benefit is confirmed more consistently for mental compared to psychomotor development, and appears to some extent to be moderated by developmental status at 12 months. The higher benefit from treatment appears for those receiving at least 91% of the biweekly home visits and program activities on at least 67% of days on the average or 716 days over 36 months. In the context of a general developmental benefit demonstrated to be due to this program of EDI [32, 33], the difference in benefit from those receiving smaller vs. larger treatment doses is modest, about three to six points on a standardized developmental measure (M = 100, SD = 15). Variation in treatment dose was associated with child health and family sociodemographic factors as well as by trial location. In particular, more frequent use of the stimulation activities was reported by better educated mothers who had already engaged in a schedule of prenatal care and had infants who reached a higher weight in the first year.

Limitations with this research include that results may not be generalizable to other L/LMIC or to other types of EDI programs. Moreover, we do not have independent observations of the implementation of the program activities at home, either in terms of quantity or quality. Program implementation dose was measured exclusively by self-report, which might have been susceptible, for example, to recall and acquiescence biases. Direct observation, though challenging to use in this context, should be less biased. Even though this trial of EDI enrolled one of the largest samples reported in L/LMIC, the sample size is still modest. This EDI was not intended for severely impaired infants. There was a 29% loss at follow-up, which included a higher proportion of parents with better resources. Power to detect significant associations with treatment dose was quite limited despite that this trial of EDI enrolled one of the largest samples reported in L/LMIC. Although a broad range of health factors were examined for associations with treatment dose, it would be useful to learn from mothers what other factors possibly influenced their use of the stimulation activities, such as motivation, belief in their efficacy, and family support. Treatment dose had a limited effect on psychomotor development, which may reflect that the EDI was not as successful in addressing development in these domains or be due to children reaching ceiling effects of the BSID at 36 months of age.

Only a few studies had previously examined whether dose of EDI during the first three years of life is associated with developmental outcomes. Our findings are consistent with prior studies that have generally reported that children who receive more exposure to EDI, however measured, display greater improvements in their cognitive development [18, 21, 24, 25]. Although only one of these studies was conducted in a L/LMIC, this too reported modest differences on developmental outcomes associated with varying home visit dose [19]. Program implementation dose was not examined. Given the differences between the EDI programs for which treatment dose has been evaluated, countries where implemented, populations targeted, and how treatment dose has been operationalized, it is difficult to generalize from this small body of research. It is impossible yet to establish a minimum effective dose. Given the importance of determining the efficacy of EDI in L/LMIC, which depends in part on information about sufficient dose, further research on the relationship between dose and outcome is much needed. Evaluations of EDI need to include such analysis to inform setting minimal targets for effective implementation.

EDI provided via home visiting has quite consistently shown to promote development in children in L/LMIC e.g., [9–16]. Our research has added to this literature by showing that the same program can do so across quite different cultures, represented here by India, Pakistan, and Zambia [32]. Whereas the identical program was used, for example in terms of the same basic structure and developmental activities, the social process transpiring in the home visits would naturally vary as a function of the specific people engaged and their local culture. One strength of home visiting EDI is that in this manner it can be both programmatically structured yet culturally flexible.

## Conclusions

The body of research in which the current study is embedded quite consistently establishes that within an effective EDI, a higher dose is generally associated with better developmental outcomes. A large body of research indicates that EDI can improve early development of children in L/LMIC. Therefore EDI should be one approach used in L/LMIC to lay the foundation for improving longer-term outcomes of its population and interrupting intergenerational transmission of poverty [26]. Yet, for this to be successful, efforts to implement EDI for children need to ensure that program elements reach the children at the intended intensity. Groups of children at risk for receiving lower treatment dose may require special attention to ensure adequate effect.
